# Distinct Contributions of Autophagy Receptors in Measles Virus Replication

**DOI:** 10.3390/v9050123

**Published:** 2017-05-22

**Authors:** Denitsa S. Petkova, Pauline Verlhac, Aurore Rozières, Joël Baguet, Mathieu Claviere, Carole Kretz-Remy, Renaud Mahieux, Christophe Viret, Mathias Faure

**Affiliations:** 1 CIRI, International Center for Infectiology Research, Université de Lyon, 69007 Lyon, France; denitsa.petkova8@gmail.com (D.S.P.); pverlhac@gmail.com (P.V.); aurore.rozieres@inserm.fr (A.R.); joel.baguet@inserm.fr (J.B.); mathieu.claviere@inserm.fr (M.C.); renaud.mahieux@ens-lyon.fr (R.M.); christophe.viret@inserm.fr (C.V.); 2 INSERM, U1111, 69007 Lyon, France; 3 CNRS, UMR5308, 69007 Lyon, France; 4 Ecole Normale Supérieure de Lyon, 69007 Lyon, France; 5 Université Lyon 1, Centre International de Recherche en Infectiologie, Avenue Tony Garnier 69365 Lyon CEDEX 07, France; 6 Institut NeuroMyoGène, CNRS UMR5310, INSERM U1217, Université Lyon 1, F-69622 Villeurbanne, France; Université de Lyon, Lyon France; carole.kretz@univ-lyon1.fr; 7 Equipe labellisée Ligue nationale contre le cancer, France; 8 Equipe labellisée Fondation pour la Recherche Médicale FRM, France; 9 Institut Universitaire de France, France

**Keywords:** autophagosome, maturation, measles virus, autophagy receptor

## Abstract

Autophagy is a potent cell autonomous defense mechanism that engages the lysosomal pathway to fight intracellular pathogens. Several autophagy receptors can recognize invading pathogens in order to target them towards autophagy for their degradation after the fusion of pathogen-containing autophagosomes with lysosomes. However, numerous intracellular pathogens can avoid or exploit autophagy, among which is measles virus (MeV). This virus induces a complete autophagy flux, which is required to improve viral replication. We therefore asked how measles virus interferes with autophagy receptors during the course of infection. We report that in addition to NDP52/CALCOCO_2_ and OPTINEURIN/OPTN, another autophagy receptor, namely T6BP/TAXIBP1, also regulates the maturation of autophagosomes by promoting their fusion with lysosomes, independently of any infection. Surprisingly, only two of these receptors, NDP52 and T6BP, impacted measles virus replication, although independently, and possibly through physical interaction with MeV proteins. Thus, our results suggest that a restricted set of autophagosomes is selectively exploited by measles virus to replicate in the course of infection.

## 1. Introduction

To maintain their integrity, cells engage various processes including autophagy, a lysosomal-dependent catabolic process, which allows the degradation of deleterious cytoplasmic components [1]. Macroautophagy, thereafter referred to as autophagy, is particularly efficient in this function as this form of autophagy is the only one that permits the recycling of very large portions of the cytoplasm after their sequestration within de novo formed autophagosomal vesicles. Thus, among intracellular substrates, autophagosomes can surround invading intracellular pathogens to target them to the lysosomal pathway; the degradation of pathogens through the autophagy pathway is known as xenophagy [2]. However, most infectious pathogens have evolved strategies to escape autophagy or even to use some properties of this cellular mechanism to optimize their intracellular life cycle; measles virus (MeV) is a striking example of such an optimization [3,4].

MeV, which is responsible for measles, is among the most contagious human pathogens [5]. This virus first affects the respiratory tract, before disseminating within the whole body. Among measles, the clinical symptoms are a fever, cough, and generalized maculopapular rash. Moreover, one to two weeks after MeV infection, a profound immunosuppression state is established which, although transient, can lead to the establishment of secondary opportunistic infections responsible for most of the MeV infection-induced complications [6]. Despite the existence of an efficient vaccine, MeV is still responsible for a significant proportion of mortality worldwide, especially in developing countries, and recent outbreaks have highlighted the importance of better understanding how the virus deals with the human host cell defenses to establish a productive infection [5].

MeV is an enveloped virus with a negative-stranded RNA genome [7]. After entering a cell, eight viral proteins are synthetized: six structural factors, which ensure viral genome replication and new particles formation (MeV-N, MeV-P, MeV-L, MeV-M and MeV-H, and MeV-F); and two non-structural proteins, which counteract, or hijack, cellular pathways to optimize intracellular replication (MeV-V ad MeV-C). Replication and virus assembly take place within the cytosol, and newly formed infectious particles bud from the plasma membrane before secondary infections. Finally, infected cells can fuse with uninfected cells to form syncytia, allowing the virus to spread from one cell to another without virus exposition outside of the infected cells.

Our group reported that upon infection, MeV can induce autophagy through three independent pathways [3,4,8,9,10,11]. First, the engagement of CD46, one of the MeV cell surface receptors, induces autophagy upon virus entry: this pathway only concerns attenuated/vaccinal strains of MeV, which use CD46 to infect cells [4,10,11,12]. A few hours post infection, a second signaling pathway leads to autophagy induction following the expression of MeV-C and its interaction with the autophagy-regulating protein IRGM (Immunity-Related GTPase family M protein) [3,8,9]. Finally, cell-cell fusion can also trigger autophagy, which contributes to sustaining both infected-syncytia viability and MeV replication [4]. Thus, MeV displays a very intricate relationship with autophagy and benefits from this process, only if completed, in order to efficiently produce new infectious particles. However, it remains to be understood how MeV escapes from autophagy degradation, especially in regards to its putative detection by autophagy receptors, whose function is to transfer pathogens to the autophagy machinery for degradation.

Autophagy receptors have the ability to bind intracellular pathogens or components of these pathogens and to target them toward growing autophagosomes. To this end, autophagy receptors contain LC3 interacting regions (LIR) in their primary sequence that are able to bind-members of the *autophagy-related* protein 8 (ATG8) family (LC3 and GABARAP (gamma-aminobutyric acid receptor-associated protein family) members in mammals), and which are essential factors anchored in the membrane of phagophores in order to drive autophagosome formation [13]. Among autophagy receptors, NDP52, optineurin (OPTN), and T6BP were concomitantly associated with the biogenesis of phagophores [14]. Independently of this role, we recently reported that NDP52 and OPTN can ensure the maturation of autophagosomes by themselves, resulting in the fusion of autophagosomes with lysosomes [15,16]. Thus, during xenophagy, NDP52 and OPTN can play a dual function: (i) they function as autophagy receptors that target pathogens to autophagy; and (ii) they also behave as autophagy adaptors that regulate autophagosome-lysosome fusion in order to degrade the entrapped pathogens. T6BP is another autophagy receptor which might have such a dual function, as recently reported in the context of a bacterial infection [17].

The role and regulation of autophagosome maturation during infection remain poorly understood. Moreover, although the role of autophagy receptors has been widely studied in the context of bacterial infection, little is known in relation to their functions upon viral infections. Since a complete autophagy flux is necessary for efficient MeV replication, we investigated the question of the requirement of autophagy receptors in autophagosome maturation and MeV replication.

## 2. Results

### 2.1. T6BP Promotes Autophagosome Maturation

**Figure 1:**
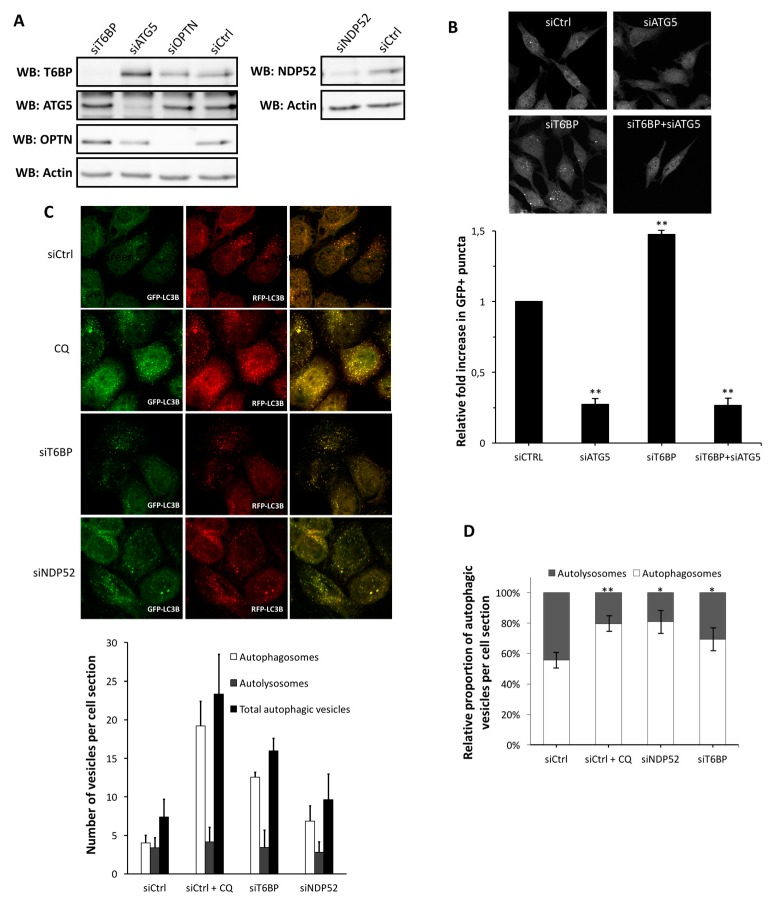
T6BP function in autophagosome maturation. (**A**) HeLa cells transfected with the indicated short interfering RNAs (siRNAs) for 48 h, were lysed, and the expression of relevant proteins was probed by Western blotting; (**B**) GFP-LC3 HeLa cells were transfected or co-transfected with the indicated siRNAs for 48 h and fixed for analysis by confocal microscopy. Representative profiles are shown along with a graph expressing the relative fold induction of the dot number compared with control cells; (**C**) mRFP-GFP-LC3 HeLa cells were transfected with the indicated siRNAs for 48 h and were treated or not treated during the last 2 h of culture with chloroquine. Representative profiles of autophagosomes (RFP^+^GFP^+^ dots) and autolysosomes (RFP^+^GFP^−^ dots) per cell section assessed by confocal microscopy are shown and were quantified. Results are expressed as absolute numbers of individual vesicles (total autophagic vesicles = all RFP^+^ dots); (**D**) Results in (**C**) are shown as the percentage of total autophagic vesicles; (**B**,**C**) were each carried out three times in duplicates. GFP: green fluorescent protein; RFP: red fluorescent protein; WB: Western blot; Ctrl: control; CQ: chloroquine.

We recently reported that NDP52 and OPTN can both regulate the maturation of autophagosomes, which can contribute to the control of intracellular bacterial growth [15,16]. Since T6BP was recently shown to contribute to the efficient autophagy-mediated clearance of bacteria [17], we started by investigating a potential role for this receptor in autophagosome maturation. To this end, we first used GFP-LC3-HeLa cells, allowing for the quantification of autophagosomes by confocal microscopy through the visualization of green fluorescent protein positive (GFP^+^) dots [18]. Interestingly, we found that the reduced expression of T6BP using specific short interfering RNA (siRNA) (Figure 1A) led to an increased number of GFP^+^ dots (Figure 1B). These structures were most certainly autophagosomes since their accumulation was prevented by the concomitant reduction of the expression of the autophagy essential protein ATG5 (Figure 1A,B).

An increased number of autophagosomes can either result from an induction of de novo autophagosome formation, or from the prevention of the recycling of autophagosomes due to a block in the fusion between autophagosomes and lysosomes. To determine the role of T6BP in the autophagy flux, we used mRFP-GFP-LC3-HeLa cells. In these cells, the mRFP-GFP-LC3 probe allows for the discrimination between autophagosomes, which express both GFP and red fluorescent protein (RFP) fluorochromes, and are therefore detected as yellow dots by confocal microscopy and autolysosomes in which only RFP fluoresces due to the high sensitivity of GFP to acidic environments [18]. Strikingly, when compared to control cells, we found a strong increase in the autophagosome number in siT6BP-treated cells, similarly to what we found (as expected) in siNDP52-treated cells or in chloroquine-treated cells, a drug which prevents the acidification of autolysosomes and leads to the accumulation of non-degradative autophagic vesicles (Figure 1A,C). Thus, when we analyzed the autophagosomes/autolysosomes ratio, we observed that the reduced expression of T6BP led to a significantly increased percentage of autophagosomes over autolysosomes, when compared to control cells (Figure 1D). Altogether, these results indicate that like NDP52 and OPTN [15,16], T6BP plays a role in the maturation of autophagosomes.

### 2.2. T6BP and NDP52, but Not OPTN, Are Required for MeV Replication

**Figure 2:**
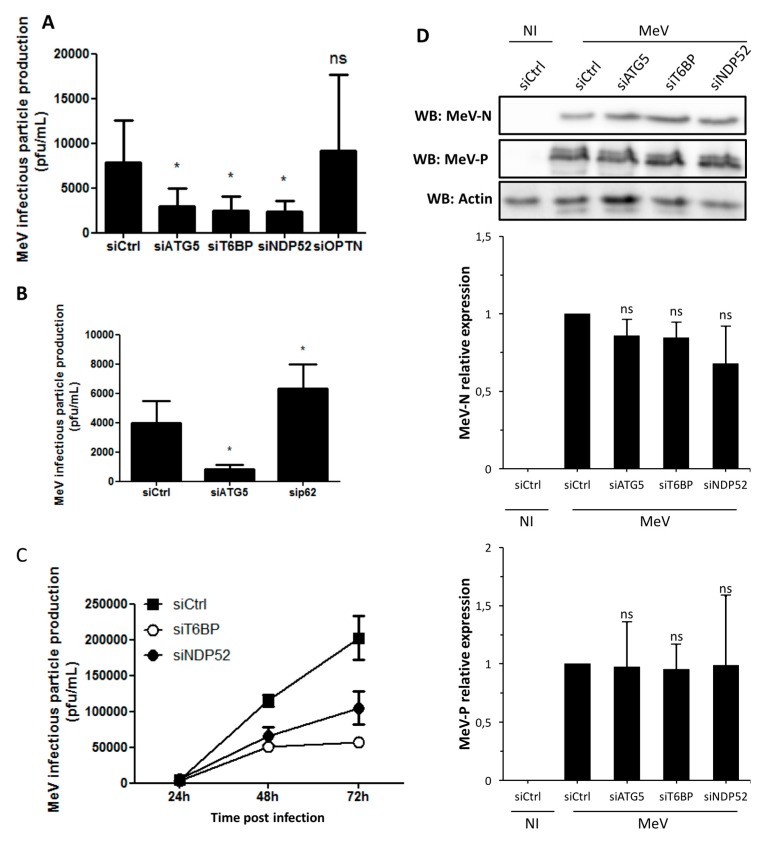
Involvement of autophagy receptors in measles virus (MeV) replication. (**A**,**B**) HeLa cells were transfected with the indicated siRNAs for 48 h, then infected with MeV (multiplicity of infection (MOI) 0.1). 48 h post infection infectious virus particles were titrated by a plaque assay; (**C**) HeLa cells were transfected with the indicated siRNA for 48 h, then infected with MeV (MOI 1). One, two, or three days post infection, infectious virus particles were titrated by a plaque assay; (**D**) Cells were treated as in (**A**). Expression of measles virus N and P proteins was assessed by Western blotting. Representative results are shown and are accompanied by a graph representing the intensity of MeV-N and MeV-P expression over Actin normalized to Control condition.(**A**,**B**,**D** error bars and mean ± SD are from three independent experiments; **C** is one experiment representative of two independent ones carried out in duplicates). NI: non infected

We previously reported that MeV infection induces a complete autophagy flux, and that full autophagosome maturation is required for an efficient MeV intracellular replication [4]. Since T6BP, NDP52, and OPTN are all individually involved in autophagosome maturation, we thought that their respective reduced expression could compromise MeV infectious particle production. To test this hypothesis, we treated cells with specific siRNAs to reduce the expression of each autophagy receptor (Figure 1A), and we measured the production of infectious MeV particles after two days of infection. First, as control, we treated cells with siATG5 which, as expected, compromised the replication of MeV (Figure 2A). Interestingly, the absence of either T6BP or NDP52 also strongly reduced the ability of MeV to produce infectious particles in infected cells (Figure 2A). This was not due to a reduced level of virus entry since neither T6BP nor NDP52 silencing impacted the high expression level of CD46 (data not shown), which is the MeV receptor involved under these conditions. Surprisingly, not every autophagy receptor involved in autophagosome maturation impacted viral replication: indeed, the extinction of OPTN did not prevent efficient MeV replication (Figure 2A). Interestingly, the silencing of the autophagy receptor p62/SQSTM1 did not prevent MeV replication, but instead, facilitated the replication of the virus (Figure 2B). This result suggests an anti-MeV function for the autophagy receptor p62, which plays no role in autophagosome maturation [15]. We further confirmed the importance of the expression of T6BP or NDP52 during MeV replication, by showing that the reduced expression of any of these proteins significantly prevented the replication of MeV from one to three days of infection (Figure 2C). Nevertheless, similarly to what we previously observed in autophagy-defective or autophagosome maturation-defective MeV-infected cells [4], the level of expression of two viral structural proteins, MeV-N and MeV-P, were not significantly affected by the reduced expression of either T6BP or NDP52 (Figure 2D). These data suggest that autophagy intervenes in MeV replication in a step downstream of viral protein synthesis. Together, these results suggest that MeV differentially uses the autophagy receptors involved in autophagosome maturation to efficiently replicate.

### 2.3. NF-κB Independent role of T6BP and NDP52 in MeV Replication

**Figure 3:**
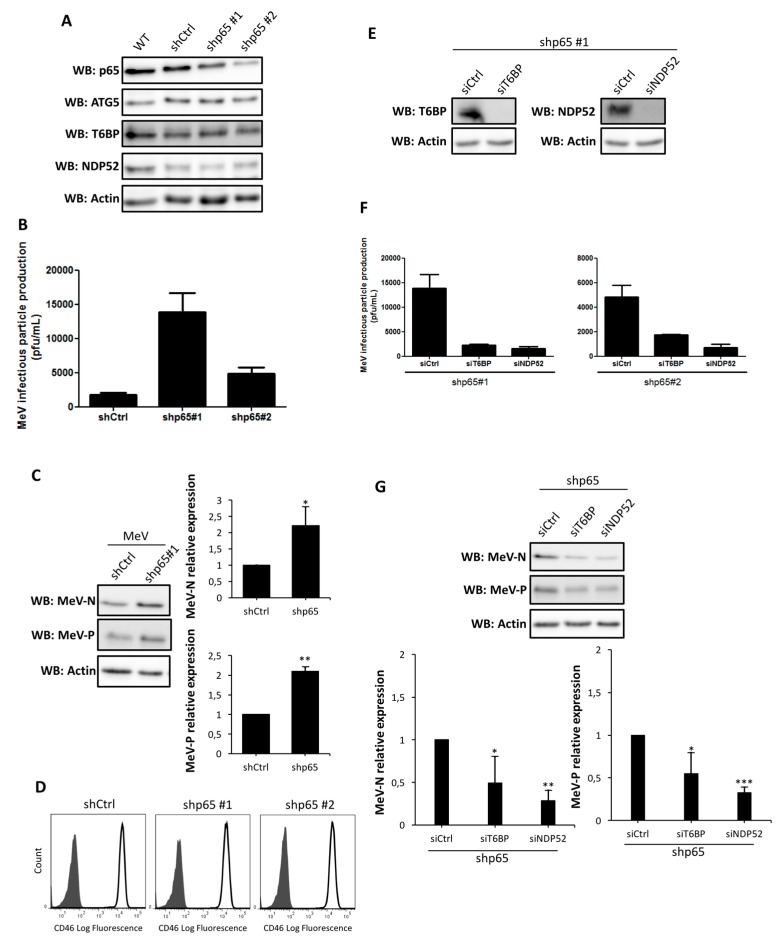
NF-κB-independent role of T6BP and NDP52 in MeV replication. (**A**) p65/RelA-expressing HeLa cells and shControl-expressing HeLa cells were transfected with the indicated siRNAs for 48 h, then lysed, and the expression of relevant proteins was probed by Western blot; (**B**) p65/RelA-expressing HeLa cells and shControl-expressing cells were infected with MeV (MOI 0.1). 48 h post infection, infectious virus particles were titrated by a plaque assay; (**C**) Cells from (**B**) were lysed 48 h post infection. Expression of measles virus N and P proteins were assessed by Western blotting. Representative results from shp65#1 are shown and are accompanied by a graph representing the intensity of MeV-N and MeV-P expression over Actin normalized to shControl-expressing cells condition. Means ± SD of four independent experiments are represented (two with the shp65#1 cell line and two with the shp65#2 cell line); (**D**) p65/RelA-expressing HeLa cells and shControl-expressing HeLa cells were stained for CD46 expression and analyzed by flow cytometry; grey histograms = isotype control, white histograms = CD46 labelling. (**E**–**G**) p65/RelA-expressing HeLa cells were treated with indicated siRNAs for 48 h; (**E**) Cells were lysed and the expression of relevant proteins was probed by Western blotting. Results regarding cell line shp65 #1 are represented. Similar results were obtained with shp65 #2. Cells were infected with MeV (MOI 0.1) and 48 h post infection, infectious virus particles were titrated by a plaque assay (**F**) or lysed; (**G**) Expression of measles virus MeV-N and MeV-P proteins was assessed by Western blotting. Representative results are shown and are accompanied by a graph representing the intensity of measles proteins expression over Actin normalized to control siRNA condition; (**B**,**F**) Means ± SD of one representative experiment out of two independent ones carried out with each shp65/RelA-expressing cell line in duplicates; (**G**) Means ± SD of four independent experiments.

By interacting with the deubiquitinase A20, T6BP has been reported to serve as an intermediate in order to dampen the activation of the nuclear factor kappa-light-chain-enhancer of activated B cells (NF-κB) signaling pathway [19]. Similarly, NDP52 was also reported to negatively impact the NF-κB signaling pathway [20]. Since the activation of the NF-κB signaling pathway could lead to the control of MeV infection [21], we thought that the decreased MeV replication observed in siT6BP-treated cells could result from an upregulation of NF-κB activity, independently of its function in autophagosome maturation. To explore this possibility, we used HeLa cells stably expressing shRNA targeting the NF-κB essential component p65/RelA of the canonical pathway (Figure 3A); these cells have a strongly compromised NF-κB activity [22,23]. We tested two shp65/RelA-expressing clones and found that both expressed equivalent amounts of ATG5, T6BP, or NDP52 compared to shControl-expressing clones (Figure 3A). Nevertheless, we found that the two NF-κB-defective HeLa clones were much more efficient in supporting MeV infectious particle production after two days of infection when compared with shControl-expressing HeLa cells (Figure 3B). We confirmed this observation by looking at the level of expression of two MeV proteins in the course of infection, MeV-N and MeV-P, which were both more expressed in shp65/RelA-expressing infected cells than in control infected cells (Figure 3C). The ability of shp65/RelA-expressing HeLa cells to sustain an efficient MeV replication was not due to a better infection of these cells since we observed an equivalent level of expression of the MeV cell surface receptor CD46 on shp65/RelA- and shControl-expressing cells (Figure 3D). Together, these results suggest that the NF-κB signaling pathway in HeLa cells could contribute to partially controlling intracellular MeV replication, possibly by controlling events upstream of viral protein translation.

To determine whether T6BP or NDP52 have a NF-κB independent role in MeV replication, we infected shp65/RelA-expressing cells, in which we reduced the expression of either T6BP or NDP52 using specific siRNAs (Figure 3E). Interestingly, the absence of any of these proteins in cells defective for the NF-κB signaling pathway negatively impacted the production of infectious MeV particles (Figure 3F). Moreover, the levels of expression of both MeV-N and MeV-P were also reduced in shp65/RelA-expressing cells, exhibiting a reduced expression of T6BP or NDP52 (Figure 3G). Therefore, altogether, these results indicate that the absence of T6BP or NDP52 can negatively impact the replication of MeV, independently of their potential role in the NF-κB signaling pathway.

### 2.4. T6BP and NDP52 Can both Interact with MeV Proteins

**Figure 4:**
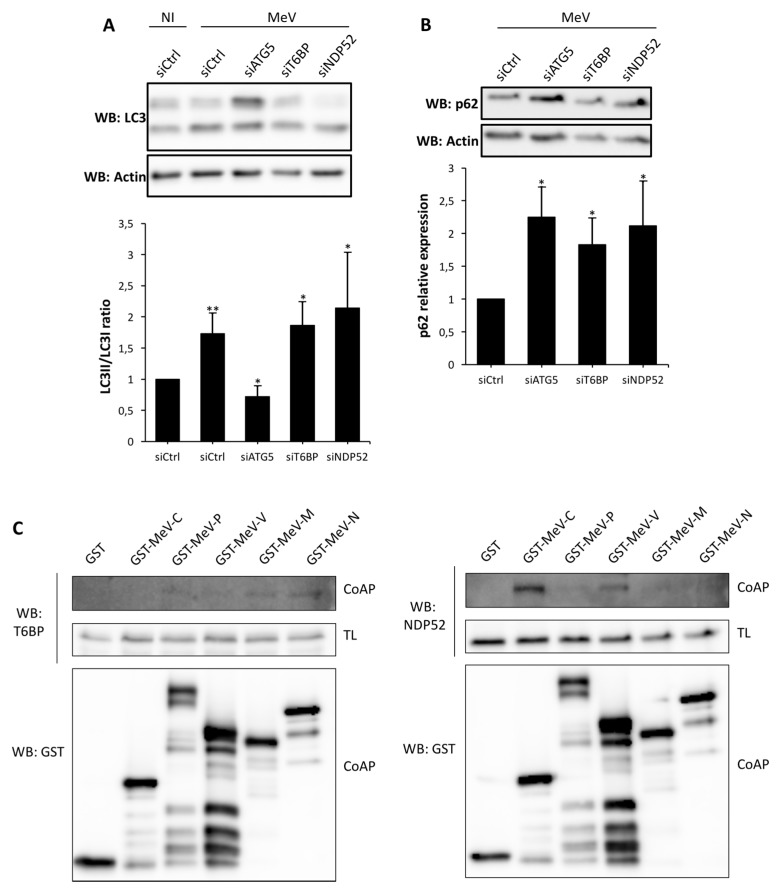
MeV protein interactions with NDP52 and T6BP. (**A**) HeLa cells were transfected with the indicated siRNAs for 48 h, and infected or not infected with MeV (MOI 0.1). 48 h post infection, cells were lysed, and anti-LC3 and anti-Actin Western blots were performed. Representative results are shown along with a graph representing the intensity of LC3 II/LC3 I bands normalized to the uninfected control condition; (**B**) HeLa cells were transfected with the indicated siRNAs for 48 h and infected with MeV (MOI 0.1). 48 h post infection, cells were lysed, and anti-p62 and anti-Actin Western blots were performed. Representative results are shown along with a graph representing the intensity of p62/Actin bands normalized to the control condition; (**A**,**B**) Means ± SD of three independent experiments are represented; (**C**) Cells were transfected with vectors encoding the indicated viral protein. Two days later, cells were lysed and GST-tagged proteins precipitated and proteins were blotted for endogenous T6BP or NDP52 as indicated. Co-AP: co affinity precipitation; TL: total lysate, GST: glutathione S-transferase.

To further depict the role of T6BP and NDP52 in MeV replication, we then asked whether the reduced expression of T6BP or NDP52 impacted MeV-induced autophagy. To this end, we looked at the conversion of LC3-I into LC3-II by Western blotting, which is a hallmark of autophagy modulation indicative of an increase in the number of autophagosomal vesicles [18]. As expected, MeV infection led to an increased level of the expression of LC3-II in control cells that was not detected in siATG5-treated MeV-infected cells (Figure 4A). However, in cells with a reduced expression of either T6BP or NDP52, MeV infection still led to an increase in LC3-II expression, suggesting that autophagy was still modulated in these cells (Figure 4A). This increase in LC3-II could, however, result from the impact of the absence of the autophagy receptors on the autophagy flux, as described above, independently of MeV infection. Furthermore, as expected, when autophagy was completely prevented by using siATG5, MeV infection led to the accumulation of p62. Indeed, p62 is an autophagy receptor which is also a main endogenous substrate of autophagy and accumulates in cells defective for autophagy. Interestingly, we obtained similar results when reducing the expression of T6BP or NDP52 (Figure 4B).

To determine whether T6BP and NDP52 could interact with MeV proteins, we tested whether overexpressed MeV proteins could co-immunoprecipitate with endogenous T6BP or endogenous NDP52 in mammalian cells. As shown in Figure 4C, we found that T6BP can interact with MeV-N, whereas NDP52 can interact with MeV-C or MeV-V. These results suggest that T6BP and NDP52 could interact with MeV proteins during the course of the infection and thereby, contribute to facilitate MeV replication through the modulation of autophagosome maturation.

### 2.5. Independent Contribution of T6BP and NDP52 in MeV Replication

**Figure 5:**
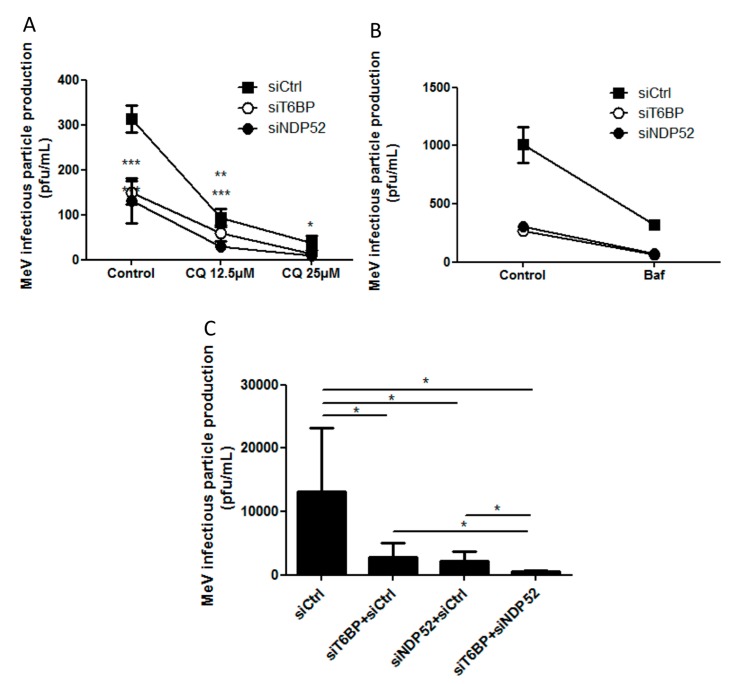
Autophagosome maturation and MeV replication. (**A**,**B**) HeLa cells were transfected with the indicated siRNAs for 48 h. Cells were then simultaneously infected with MeV (MOI 0.1) and treated or not treated with 25 µM or 12.5 µM of Chloroquine (**A**) or 25 nM of Bafilomycin A1 (Baf A1) (**B**) 48 h post infection and drug treatment, infectious virus particles were titrated by a plaque assay; (**C**) HeLa cells were transfected or co-transfected with the indicated siRNAs for 48 h. Cells were infected with MeV (MOI 0.1) and 48 h post infection, infectious virus particles were titrated by a plaque assay. Means ± SD of three independent experiments performed in duplicates are represented; (**A**,**C**), means ± SD of three to four independent experiments performed in duplicates; (**B**) means ± SD of one representative experiment out of four independent ones.

To determine whether the usage of the autophagy receptors T6BP and NDP52 in autophagosome maturation during MeV infection is a rare or a frequent event, we looked at the impact of these receptors on MeV replication in the context of a partial inhibition of pan-autophagosome-maturation. We reasoned that if those events were rare, the extinction of expression of either T6BP or NDP52 concomitantly with a moderate concentration of pan-autophagosome maturation inhibitors, would not significantly further impact MeV replication. To this end, we used non-saturating concentrations of chloroquine to partially prevent the autophagy flux in order to limit the prevention of MeV replication. Indeed, whereas 50 µM of chloroquine completely abolished MeV replication, 25 µM and 12.5 µM concentrations inhibited MeV replication by 90% and 70%, respectively (Figure 5A and not shown). Interestingly, we found that with such chloroquine concentrations, the reduced expression of either T6BP or NDP52 significantly prevented the further replication of MeV, compared to chloroquine only-treated cells (Figure 5A), without altering the cellular viability (not shown). Similar results were found during infection, when we used another inhibitor of the autophagy flux at a non-saturating concentration, Bafilomycin A1 (25 nM, inhibition of MeV replication by 60%, Figure 5B). Thus, these results indicated that the maturation of autophagosomes, supported either by T6BP or NDP52, can further prevent the reduced MeV replication imposed by drugs which randomly block the maturation of all autophagosomes, suggesting that the regulation of autophagosome maturation by T6BP and NDP52 is not a rare event and is important for MeV replication.

We then asked whether T6BP and NDP52 could regulate the maturation of identical or distinct autophagosomes induced in the course of MeV infection. If each autophagosome requires both T6BP and NDP52 to maturate, we reasoned that the concomitant extinction of the two proteins would not further impact the replication of MeV, when compared to the respective single reduced expression of each receptor. Interestingly, we found that the concomitant reduced expression of T6BP and NDP52 has a significantly stronger inhibitory potential on MeV replication than the individually reduced expression of these proteins (Figure 5C), without altering the cellular viability (not shown). These results strongly suggest that T6BP and NDP52 could regulate the maturation of distinct autophagosomes, which are both required for an optimal MeV replication.

## 3. Discussion

In the course of infection, viruses have to face cellular immune protection mechanisms [24]. Among them, viral components can be detected by autophagy receptors and degraded through the lysosomal pathway to fight viral infection [25,26,27,28,29]. However, MeV infection, although inducing a complete and productive autophagy flux, meaning from the formation of an isolated phagophore to the degradation and recycling of autophagy substrates by autolysosomes, seems insensitive to autophagy, but instead, uses this process for an optimal replication [3,4]. Here, we report that autophagy receptors, which also play an important role in the maturation process of autophagosome-lysosome fusion, are not used equivalently by MeV during cell infection.

Autophagy receptors ensure the recognition of cytosolic substrates to target them to the autophagy machinery [30]. We recently reported that the two autophagy receptors NDP52 and OPTN also regulate the fusion between autophagosomes and lysosomes, and therefore, play the dual role of autophagy receptors and autophagy adaptors, for an efficient degradation by autophagy [15,16]. Here, we extend this discovery to T6BP, which also regulates the maturation of autophagosomes, since the reduced expression of this protein led to an accumulation of autophagosomes. T6BP (also called TAXIBP1 or CALCOCO_3_) has several homologies with NDP52 (also called CALCOCO_2_), which might explain how this protein contributes to autophagy maturation. NDP52 contains a MYOSIN VI binding domain and a LIR motif, which interact with MYOSIN VI and LC3B, respectively [15,31]. These two binding sites were shown to be essential for NDP52-mediated autophagosome maturation [15]. Indeed, MYOSIN VI interacts with the endosomal protein TOM-1 [14], and LC3B is anchored in the autophagosomal membrane. By interacting with MYOSIN VI and LC3B, NDP52 connects the autophagosome with the endosomal pathway. Similarly to NDP52, T6BP was also reported for its potency to physically bind MYOSIN VI via two essential residues, C688 and C715 [30]. T6BP also contains a LIR domain, allowing its co-localisation and interaction with LC3B [17,32]. Thus, through the concomitant interaction with MYOSIN VI and LC3B, T6BP could govern the maturation of autophagosomes, similarly to NDP52. Indeed, a recent work described the essential role for both T6BP and MYOSIN VI in the late phase of autophagy for an efficient clearing of intracellular infection by *Salmonella typhimurium* [17]. Both T6BP and NDP52, but not OPTN, contain a so-called SKICH domain whose function is undetermined. Whether this domain plays a role in the differential impact of these receptors on MeV replication remains to be studied.

T6BP and NDP52 are both involved in a negative regulation of the canonical NF-κB signaling pathway [19,20]. Since viral infections can be regulated by the NF-κB pathway [21], the impact of the reduced expression of T6BP or NDP52 on MeV replication could have been due to its role in an autophagy-independent mechanism. However, the use of NF-κB defective cells allowed us to demonstrate that the absence of T6BP or NDP52 impacted MeV replication, independently of the activation of this transcription factor. Thus, although we cannot exclude that a T6BP/NDP52-dependent regulation of NF-κB could contribute to the partial control of MeV replication, the role of these autophagy receptors in the maturation of autophagosomes appears to be predominantly required for an efficient MeV replication. OPTN has also been reported to either positively or negatively regulate the NF-κB signaling pathway [33,34]. The fact that siOPTN did not affect MeV replication also suggests that the potential role of T6BP and NDP52 in NF-κB signaling has no significant role in the course of MeV infection in HeLa cells.

**Figure 6:**
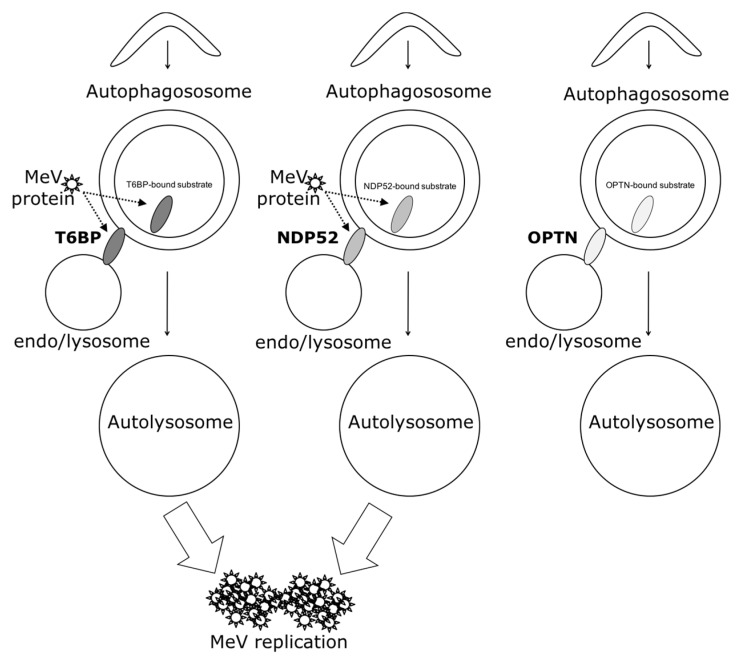
Schematic model of the interplay of autophagy receptors with MeV replication. NDP52, OPTN, and T6BP could all play a dual function in autophagy: to target selective substrates towards autophagosomes and to regulate substrate-containing autophagosome maturation (which could be those for which they targeted selective substrates), for an efficient degradation. Only autophagosome maturated via an NDP52 or T6BP pathway are exploited by MeV to improve its replication. Such exploitation could occur via the usage of each receptor for their functions in the targeting and/or the maturation processes (dashed arrows).

In our work, several lines of evidence suggest that autophagosome maturation could be regulated independently by each autophagy receptor/adaptor and that they could be exploited individually by MeV to replicate. As described, beyond their function as autophagy receptors, NDP52, T6BP, and OPTN also have a role in the maturation of autophagosomes. Strikingly, the reduced expression of OPTN did not impact the production of infectious MeV particles, contrary to the reduced expression of T6BP or NDP52. Thus, not all maturated autophagosomes seem to be involved in MeV replication (e.g., the ones regulated by OPTN), but only some of them, such as those regulated by NDP52 or T6BP. How MeV makes the distinction between individual autophagosomes remains to be fully depicted. This could occur through the interaction of viral proteins with either NDP52 or T6BP, which could potentiate the maturation of autophagosomes regulated by these two proteins. Indeed, we found that distinct MeV proteins have the ability to interact with either NDP52 or T6BP, but whether such interactions take place in the course of infection and drive autophagosome maturation remains to be fully investigated. Alternatively, T6BP and NDP52, but not OPTN, could target selective substrates to autophagy whose degradation is required for MeV replication (Figure 6). Since MeV-induced autophagy contributes to delay the death of infected cells [4], such selective substrates could be infection-induced apoptotic factors, but this also needs to be analyzed.

Another piece of evidence suggesting a distinct usage of individual NDP52-mediated and T6BP-mediated autophagosome maturation for MeV replication is the fact that the co-reduced expression of NDP52 and T6BP impacted MeV replication more efficiently than their individual reduction. If NDP52 or T6BP were both co-engaged in the maturation process of all autophagosomes, their single reduction would have impacted the function of both receptors on MeV replication. Although used at saturating concentrations, we cannot exclude that siRNA treatments are not completely efficient at reducing the expression of individual proteins. Thus, siNDP52 (or siT6BP) could permit some NDP52/T6BP-dependent autophagosome maturation, which would not have been impacted due to residual endogenous NDP52 upon siNDP52 treatment (or T6BP upon siT6BP treatment). However, if all autophagy receptors were engaged in the regulation of each individual autophagosome, we would have expected that the reduced expression of OPTN, which also impacts autophagosome maturation, would have decreased MeV replication. Thus, although we cannot exclude that a unique molecular machinery involving NDP52, T6BP, and OPTN is required for the maturation of autophagosomes, our results suggest a very fine tuned molecular regulation of autophagosome maturation, which could be exploited by MeV to replicate (Figure 6).

In the course of infection, the interplay of MeV with the autophagy process is very intricate, as we already reported that several signaling pathways are involved. The deeper study of this specific host-pathogen interaction allowed us to reveal here a potential individual regulation of autophagosome maturation by individual autophagy receptors. Our study offers interesting perspectives in regards to both the understanding of autophagy molecular regulation, a cellular process whose deregulation is associated with several human pathologies, and the potential development of strategies to fight MeV infection, one of the most contagious human diseases, possibly by targeting individual molecules involved in the specific maturation of autophagosomes, without altering the complete autophagy process.

## 4. Experimental Procedures

### 4.1. Antibodies and Reagents

Antibodies used were: anti-T6BP (HPA024432) anti-NDP52 (HPA023195), anti-LC3B (L7543), anti-actin (A2066), and anti-ATG5 (A0856), all from Sigma-Aldrich, anti-OPTN (Abcam, ab23666 Paris, France), anti-SQSTM1/p62 (Santa Cruz Biotechnology, sc-28359, Heidelberg, Germany), and anti-p65/RelA (Millipore #06-418, Molsheim, France). Anti-MeV-N (mouse monoclonal, clone 120) and anti-MeV-P (rabbit polyclonal, clone J37171) were used. Anti-CD46 conjugated to the PE antibody (8E2 clone) was from ThermoFisher Scientific (12-0469-42, Courtaboeuf, France). Secondary antibodies used were: anti-Mouse conjugated to Peroxydase (A2304) from Sigma-Aldrich and anti-Rabbit conjugated to HRP (NA9340). Pharmacological agents used were Bafilomycin A1 (25 nM) (InvivoGen #tlrl-baf1, Toulouse, France) and Chloroquine (25 µM or 12.5 µM) (C6628, Sigma-Aldrich, St. Quentin Fallavier, France).

### 4.2. Cell Culture

HeLa, GFP-LC3-HeLa, mRFP-GFP-LC3-HeLa, shp65-HeLa, and Vero cells were maintained in DMEM medium, supplemented with 10%FBS, 0.1% Gentamicin. An additional 500 µg/mL of Geneticin/G418 was added for GFP-LC3-HeLa, mRFP-GFP-LC3-HeLa, and shp65-HeLa cell cultures. The shCtrl, shp65#1, and shp65#2 HeLa cell lines used in this study were the HeLa-cont#1, HeLa-p65 KD#1, and HeLa-p65 KD#2 cell lines used in [21], respectively.

### 4.3. siRNA Transfection

The day before transfection with siRNA, the cells were seeded in six-well plates with 1 × 10^5^ cells per well in OPTIMEM complemented with 10% fetal bovine serum (FBS), 2 mM of l-glutamine, 50 mg/mL of Gentamycin, 0.1 mM non-essential amino acid, 0.1 mM pyruvate sodium, and 0.1 g/L bicarbonate sodium. The cells were transfected with 100 pmol of total siRNA using Lipofectamine RNAiMAX from Invitrogen (13778-150, Courtaboeuf, France), according to the manufacturer’s instructions. Protein expression level was assessed by Western blotting four days post transfection (lysis buffer: PBS 1X, 0.5% Nonidet P40, and protease inhibitor) (Complete Mini EDTA free, Roche Applied Science #04693159001, Meylan, France).

For titration experiments, the cells were transferred 48h after siRNA transfection to a 24-well plate at 2 × 10^4^ cells per well. Five hours after the transfer, cells were infected with MeV.

### 4.4. MeV Strains and Titration by Plaque Assay

Measles Virus Edmonston strain (MeV) was obtained from ATCC. HeLa cells were infected with MeV at the indicated MOI. After the indicated period of infection, cells were submitted to five freeze (−80 °C)-thaw cycles (ambient temperature) and infectious viral particles were quantified by limiting dilution on confluent Vero cells. Briefly, supernatants were diluted in DMEM culture medium with 2% FBS. The dilutions covered the range from 1/2 to 1/810 and each dilution was tested in duplicate. A total of 0.45 mL of each dilution was loaded onto the Vero cell monolayer. After 1.5 h of adsorption at 37 °C, 800 µL of DMEM culture medium with 2% FBS was added and cells were further incubated at 37 °C, 5% CO_2_ for 48 h. Then, Vero cells were fixed and stained with Methylene Blue. Plaque-forming units (pfu) were numerated after the cell layers had been washed and left to dry. Only dilutions which displayed at least 10 pfu were taken into account. At least three dilutions were considered when calculating the viral titers for each duplicate in each experiment and the mean of the duplicates was calculated. Results are represented as a fold increase normalized to the control condition relevant to a given experiment.

### 4.5. Molecular Cloning

For mammalian cell expression, viral proteins were engineered into a pDEST27 plasmid, allowing the expression of Glutathione S-transferase (GST) tagged proteins for co-affinity purification experiments.

### 4.6. GST Co-Affinity Purification Assays

HeLa cells were seeded at 2.5 × 10^5^ cells per well in six-well plates. Twenty-four hours later, cells were transfected with 2 μg/well of plasmid encoding the GST-tagged genes. The cells were harvested 48 h late and lysed in PBS 1×, containing Calcium and Magnesium with 0.5% of Nonidet P40 and protease inhibitor cocktail (Complete Mini EDTA free, Roche Applied Science #04693159001, Meylan, France). The purified lysate was incubated overnight at 4 °C on Glutathione Sepharose beads (GE Healthcare #17-0746-01, Courtboeuf, France). Elution and Western blotting were performed the next day.

### 4.7. Confocal Microscopy

All images were taken on a confocal Zeiss LSM 710 (Marly le Roi, France) with a plan apochromat 40× objective. The quantification of fluorescent vesicles was carried out using ImageJ. The cells were cultured in 24 well-plates with a sterile coverslip in each well. The cells were fixed in ice cold acetone. At least 100 cells per individual experiment were numerated.

### 4.8. Immunofluorescence-coupled Flow Cytometry

For CD46 staining, 0.25–0.5 × 10^6^ cells were incubated in microtiter U-bottom plates with saturating concentrations of labeled monoclonal antibody (mAb) in 20 μL PBS 2% FCS/0.1% NaN3for 30 min on ice. Cells were washed twice and analyzed immediately, without fixation. The anti-human CD46 mAb used was the phycoerythrin (PE)-conjugated 8E2 clone (mouse IgG1κ) from eBioscience. A LSRII flow cytometer (Becton Dickinson, Pont-de-Claix, France) and the FlowJo software (Tristar, Ashland OR, USA) were used to collect and analyze the data. Nonviable cells were excluded using forward and side scatter electronic gating. In some experiments, results were confirmed by using the fluorescein isothiocyanate (FITC)-conjugated anti-human CD46 E4.3 mAb (mouse IgG2aκ) from BD Biosciences (Pont-de-Claix, France).

### 4.9. Statistical Analysis

All *p*-values were calculated using a one-tailed Welch’s *t*-test (Student’s *t*-test assuming non-equal variances of the samples), except for the result of Figure 5A,B for which an Anova2 Bonferroni *post hoc* test was applied; * *p* < 0.05, ** *p* < 0.01, *** *p* < 0.001.
